# Comparison of Miniaturized Raman Spectrometers for Discrimination of Carotenoids of Halophilic Microorganisms

**DOI:** 10.3389/fmicb.2019.01155

**Published:** 2019-05-29

**Authors:** Jan Jehlička, Adam Culka, Lily Mana, Aharon Oren

**Affiliations:** ^1^Faculty of Science, Institute of Geochemistry, Mineralogy and Mineral Resources, Charles University, Prague, Czechia; ^2^The Institute of Life Sciences, The Hebrew University of Jerusalem, Jerusalem, Israel

**Keywords:** portable Raman spectrometers, portable sequentially shifted excitation Raman spectrometer, halophiles, *Salinibacter*, *Corynebacterium*, *Halorubrum*, *Halobacterium*, exobiology

## Abstract

We present a comparison of the performance of four miniature portable Raman spectrometers for the discrimination of carotenoids in samples of carotene-producing microorganisms. Two spectrometers using a green laser allowing to obtain Resonance Raman (or pre-Resonance Raman) signals, one instrument with a 785 nm laser, and a recently developed Portable Sequentially Shifted Excitation Raman spectrometer (PSSERS) were used for identifying major pigments of different halophilic (genera *Halobacterium*, *Halorubrum*, *Haloarcula*, *Salinibacter*, *Ectothiorhodospira*, *Dunaliella*) and non-halophilic microorganisms (*Micrococcus luteus*, *Corynebacterium glutamicum*). Using all the tested instruments including the PSSERS, strong carotenoids signals corresponding to the stretching vibrations in the polyene chain and in-plane rocking modes of the attached CH_3_ groups were found at the correct positions. Raman spectra of carotenoids can be obtained from different types of microbiological samples (wet pellets, lyophilized culture biomass and pigment extracts in organic solvents), and can be collected fast and without time-consuming procedures.

## Introduction

Raman spectra of microorganisms can tell us about the presence of key biomarkers playing different roles for energy recovery and microorganisms survival. Detecting carotenoids or even their discrimination through Resonance Raman spectroscopy using green excitation is widely used (Marshall et al., 2007; Jehlička et al., 2014a). More advanced approaches allow obtaining information *in vivo* in the frame of individual cells (Heraud et al., 2007; Ota et al., 2018). Raman mapping of pigment distribution is also currently used (i.e., Coherent Anti-Stokes Raman Scattering microscopy, CARS microscopy, Venckus et al., 2018). Raman microspectrometry may be advantageous to learn about colonization of more or less extreme environments such as the dry Atacama or the Mojave Desert (Vítek et al., 2012) or biofilms on limestones in Europe (Storme et al., 2015). Raman spectra of carotenoids of different taxonomic groups of cyanobacteria, Archaea or algae were also recorded from specimens grown in laboratory cultures (Jehlička et al., 2014a; Kumar et al., 2015; Stoeckel et al., 2015). Other pigments were investigated as well using Raman spectroscopy. Examples are prodigiosin from *Serratia marcescens* (Jehlička et al., 2016), violacein from *Chromobacterium violaceum* (Jehlička et al., 2015), different pigments of cyanobacteria, parietin and other anthraquinones of lichens (Edwards et al., 1997, 2000; Villar et al., 2005) and astaxanthin from snow algae (Osterrothová et al., 2019). Interesting polyenic and non-polyenic pigments and acids were found using Raman microspectrometry in basidiomycetes (Tauber et al., 2018).

Discrimination of carotenoids detected using Resonance Raman spectroscopy can be challenging, mainly when they occur in mixtures in biomass. Evaluation of the presence of carotenoids of similar length of the polyenic chain can also be tricky (Jehlička et al., 2014a; Oren et al., 2018). The possibilities and limitations of Raman spectroscopy for non-destructive analysis of pigments and metabolites in different organisms were highlighted in review papers (Baranska et al., 2013; Jehlička and Oren, 2013; Jehlička et al., 2014b).

Raman spectroscopy has been applied to the study of different pigmented halophilic microorganisms (Marshall et al., 2006; Fendrihan et al., 2009; Jehlička et al., 2013). Raman spectra of pigments of halophilic organisms were also recorded using portable instrumentation. Benthic gypsum layers from the solar salterns at Eilat (Israel) were investigated. In this case it was possible to confirm previous observations on the presence of specific carotenoids produced by halophiles under different light and salinity conditions (Jehlička and Oren, 2013). During the last decade small, portable and sometimes handheld and even palm/sized Raman spectrometers were applied in some areas. These instruments allow very fast analyses for forensic applications, chemistry or cultural heritage investigations (Vandenabeele et al., 2014). Mobile Raman spectroscopy was used to detect carotenoids in rocky matrices such as in the frame of colonizations of benthic gypsum crusts in salterns (Jehlička and Oren, 2013; Culka et al., 2014). Few studies report on the application of portable or transportable Raman devices to study pigments in microorganisms in their natural environments. In their study of crusts inhabited by endolithic cyanobacteria in the Atacama desert, Vítek et al. (2012) have shown that carotenoids can easily be detected using a semi-portative Raman spectrometer with a 532 nm laser. Additionally, red diode excitation (785 nm) permitted to show the presence of scytonemin of cyanobacteria. Miralles et al. (2012) have demonstrated how portable Raman spectrometers (785 nm) can be used for detection of biomolecules, including pigments, of lichens. The presence of fluorescence derived from other organic compounds in the matrix can be a critical issue for the detection of trace amounts of carotenoids. Discrimination of individual carotenoids can sometimes be problematic as well. This is related to the similarity of many carotenoids, especially when the length of the polyenic chain is identical. Additionally, the spectral resolution of the portable Raman systems is relatively low (∼5–10 cm^−1^) and sometimes does not allow unambiguous discrimination of similar carotenoids.

More recently, a new portable Raman system was introduced on the market allowing fluorescence mitigation or even elimination (Portable Sequentially Shifted Excitation Raman Spectrometer, PSSERS, Bravo). A relatively recent method to mitigate the fluorescence with benchtop instruments is so-called Shifted Excitation Raman Difference Spectroscopy (SERDS). In simplistic terms, this method is based on the changing of the excitation laser wavelength during Raman spectral acquisition. In Sequentially Shifted Excitation (SSE) Raman spectroscopy the diode lasers operate at different temperatures, providing slightly shifted wavelengths; in this case the location of Raman intensities in spectral space changes with the excitation wavelength, while unwanted spectral intensities corresponding to fluorescence, stray light, fixed pattern detector noise, etc., remain unchanged in spectral space. This difference allows extracting the Raman spectrum separated from the fluorescence spectrum. Bravo uses a new patented technology (SSE^TM^, patent number US8570507B1) to mitigate fluorescence. This spectrometer is equipped with two excitation lasers with wavelengths (DuoLaser^TM^) ranging from 700 to 1100 nm. Each laser is temperature-shifted over a small wavelength range; for example, the distributed Bragg reflector (DBR) diode laser emits single-mode 785 nm radiation at 25°C. A typical measurement consists of collecting Raman spectra at DBR laser temperatures of 20, 23, 26, and 29°C (i.e., four sequential excitations). This yields excitation wavelengths of 784.630, 784.852, 785.074, and 785.296 nm, respectively, and gives a constant excitation shift of 0.222 nm. When converted to wavenumbers (cm^−1^), this gives a separation of substantially 3.60 cm^−1^ between the different excitations. Once the shifted excitation Raman spectra are acquired, the Raman spectrum can be extracted using data processing as described in the above-mentioned patent.

The goal of the present study is to detect carotenoids in halophilic and other microorganisms grown under laboratory conditions, using a suite of miniature Raman instruments. In this report we used four portable miniature Raman spectrometers, including the new portable sequentially shifted excitation Raman spectrometer Bravo (PSSERS) as well as the conventional portable system Inspector Raman (785 nm) and two green laser equipped instruments RaPort (532 nm) and First Guard (532 nm). The analytical performance of the Bravo instrument was first tested for identification of carotenoids of microorganisms of different origin. Obtained data were compared to those from other light and portable Raman spectrometers. It was shown that strong carotenoid signals could also be obtained under non-resonant conditions.

## Materials and Methods

### Microbial Strains and Growth Conditions

The following microorganisms were investigated: *Halobacterium salinarum*, *Haloarcula marismortui* and *Halorubrum sodomense* (halophilic Archaea), *Salinibacter ruber, Ectothiorhodospira marismortui* (halophilic Bacteria), *Dunaliella parva* (a halophilic green alga), and *Micrococcus luteus, and Corynebacterium glutamicum* (non-halophilic and slightly halotolerant bacteria). *Halobacterium salinarum* strain R1 (DSM 671) was grown in medium containing (g/l): NaCl, 250; MgCl_2_ ⋅ 6H_2_O, 5.0; KCl, 5.0; NH_4_Cl, 5.0 and yeast extract, 1.0, pH 7.0. The medium for *Haloarcula marismortui* ATCC 43049^T^ contained: NaCl, 206; MgSO_4_ ⋅ 7H_2_O, 36; KCl, 0.37; CaCl_2_ ⋅ 2H_2_O, 0.5; MnCl_2_, 0.013; yeast extract, 5. The medium for *Halorubrum sodomense* (ATCC 33755^T^) contained: NaCl, 125; MgCl_2_.6H_2_O, 160, K_2_SO_4_, 5.0; CaCl_2_ ⋅ 2H_2_O, 0.1; Yeast extract, 1.0, casamino acids, 1.0, and soluble starch, 2.0; pH 7.0. *Salinibacter ruber* M31 (DSM 13855^T^) was grown in medium containing NaCl, 195; MgSO_4_ ⋅ 7H_2_O, 25; MgCl_2_ ⋅ 6H_2_O, 16.3; CaCl_2_ ⋅ 2H_2_O, 1.25; KCl, 5.0; NaHCO_3_, 0.25; NaBr, 0.625, and yeast extract, 1.0, pH 7.0. For the preparation of 11 of growth medium for *Ectothiorhodospira marismortui* EG-1 (DSM 4180^T^), an autoclaved solution of 100 g NaC1, 0.5 g Na_2_SO_4_, and 0.1 g Bacto yeast extract in a final volume of 900 m, was prepared, to which just before inoculation the following components were added from sterile solutions: 0.33 g/l of each KH_2_PO_4_, NH_4_Cl, KCl and MgCl_2_ ⋅ 6H_2_O, 10 ml of trace element solution (Pfennig and Lippert, 1966); 0.5 g Na-acetate ⋅ 3H_2_O; 1.5 g Na_2_CO_3_; 1 M HCl to a final pH of 6.5–6.8, and 0.33 g CaCl_2_ ⋅ 2H_2_O. Immediately before inoculation, Na_2_S was added to a final concentration of 0.5 mM. *Dunaliella* medium contained: NaCl, 56.3; KCl 0.135; MgCl_2_.6H_2_O, 11.02; MgSO_4_.7H_2_O, 13.84; CaCl_2_.2H_2_O, 0.29; KH_2_PO_4_.3H_2_O, 0.039; NaNO_3_, 1.5; Na_2_-EDTA, 0.001; Na_2_CO_3_, 0.02; Citric acid, 0.006; Fe-Citrate, 0.006; Trace elements (Mo, Zn, Mn, B, Cu), final pH 7.5.

*Micrococcus luteus* ATCC 4698^T^ and *Corynebacterium glutamicum* DSM 20300^T^ were grown in 8 g/l Nutrient Broth (Difco). All media were sterilized by autoclaving. The heterotrophic bacteria were grown in 1 l cultures with shaking in 2 l Erlenmeyer flasks (35°C for *Hbt. salinarum*, *Hrr. sodomense* and *S. ruber*, 30°C for *M. luteus* and *C. glutamicum*). *D. parva* was grown without shaking at 30°C in 100 ml portions of media in 250 ml Erlenmeyer flasks in the light (100 μmol quanta.m^−2^.s^−1^). *E. marismortui* was grown in the light (30 μmol quanta.m^−2^.s^−1^) at 35°C in completely filled 300 ml glass bottles.

Late-exponential-growth phase cultures were harvested by centrifugation (8,000 × *g*, 4°C, 20 min). Cell pellets were washed in 20 ml 25% NaCl, centrifuged, and resuspended in 20 ml 25% NaCl, and centrifuged again.

The following samples were investigated by portable Raman spectrometers:

(1) Wet pellets (material from cell pellets obtained by centrifugation was smeared onto glass slides and dried at room temperature).
(2) Lyophilized cultures (small pieces of lyophilized cell material placed on glass slides).
(3) Methanol/acetone 1:1 extracts of cells (extracted in the cold at 4°C, in a refrigerator, in the dark for 2 h) followed by centrifugation and drying of the extract under a stream of nitrogen).
(4) Bligh and Dyer extracts (the dried lower chloroform phase of the two-phase chloroform – methanol – water extraction system) (Bligh and Dyer, 1959).

### Raman Spectroscopic Instrumentation

#### First Guard

The Rigaku First Guard Raman (Rigaku Raman Technologies, Burlington, MA, United States) spectrometer weighs 2.7 kg, is a handheld instrument, and is equipped with a 532 nm a doubled Nd:YAG laser for excitation. The instrument contains a built-in resistive touch screen allowing for a range of experimental settings such as the number and duration of scans to be collected. The laser power at the sample is 18 mW (whole range is 1–60 mW). The optimized experimental setting used was 1 s scan time and 40 scans accumulated for each Raman spectrum. Spectral range is 200–3200 cm^−1^, with a spectral resolution of 10–15 cm^−1^. The instrument was calibrated using a benzonitrile standard. The spectra are saved in a binary.spc format files together with a header in plaintext containing the experimental settings and state of the spectrometer.

#### Inspector Raman

The DeltaNu Inspector Raman (DeltaNu, Inc., Laramie, WY, United States) is a handheld instrument with a weight of 1.9 kg and equipped with a 785 nm diode laser for excitation. The instrument was operated via a USB 2.0 cable connection to a laptop computer running the NuSpec control software which allows to set laser power levels at the source to five settings: low, medium-low, medium, medium-high, and high, as well as controlling the duration and number of the scans. The laser power at the sample was 30 mW. Experimental setting used were 5 s scan time and 20 scans accumulated for each Raman spectrum. The spectral range is 200–2000 cm^−1^, with a spectral resolution of 8 cm^−1^. The instrument was calibrated using a polystyrene standard. The spectra are saved in a binary.spc format files together with a header in plaintext containing the experimental settings.

#### RaPort

The EnSpectr RaPort (Enhanced Spectrometry, Inc., San José, CA, United States) is a handheld Raman spectrometer (weight 1.25 kg) using a doubled Nd:YAG laser at 532 nm for excitation. The instrument was operated via a USB 2.0 cable connection to a laptop computer. The automatic mode was selected (usually less than 1 min for a single analysis), the laser power at the sample was 6 mW (measured by the LaserCheck by Coherent, Santa Clara, CA, United States). The measurement range is wide for a portable instrument, 140–4180 cm^−1^, with a maximum spectral resolution of 7 cm^−1^. No calibration is required before measurements. The spectra are saved in plaintext in.esp files together with the experimental settings and state of the spectrometer.

#### Bravo

The Bruker Bravo PSSERS (Bruker Optik GMBH, Ettlingen, Germany) weighs 1.5 kg and is equipped with two lasers in the near infrared spectral region (785 and 853 nm) with sequentially shifted excitation in its design and operation to suppress fluorescence. The instrument was operated via a wireless (Wi-Fi) connection to a laptop computer running the OPUS control software. Laser power level cannot be adjusted, but is reported to be <100 mW. Spectra were recorded using the automated settings (usually less than 1 min for a single analysis). Spectral range is 300–3200 cm^−1^ at 10–12 cm^−1^ resolution. No calibration is required before measurements. The spectra are saved in binary ^∗^.0 files together with the experimental settings and state of the spectrometer in plaintext.

The sequentially shifted excitation (SSE) of this instrument’s operation is achieved by operating the diode lasers at different temperatures, which in turn provides minor shifts (<1 nm) in the excitation wavelengths. Non-Raman signals such as various spectral artifacts and fluorescence bands appear at fixed positions in the spectral space, while the positions of the Raman bands move slightly, based on the minor changes in excitation wavelength. This is then used to computationally “subtract” the fluorescence signal and give a clearer final Raman spectrum. This SSE is used for both lasers, each of them covering a different region in the spectral range, with some partial overlap. The final Raman spectrum produced by the instrument is therefore computed from the six “raw” spectra containing both the Raman and fluorescence bands. These raw spectra can be accessed later, as they are saved in the.0 binary files containing the spectra. This method is described in greater detail by Jehlička et al. (2017). Spectra obtained are exported and then viewed and processed in GRAMS/AI 9.1 and OPUS 7.7.23 spectroscopic software. Spectra in.esp file format are converted to other working formats using the SpectraGryph 1.2 spectroscopic software. They are presented in this study both as unmodified raw spectra and baseline-corrected spectra for detailed comparison.

### Data Treatment

Differences exist between the qualities of Raman spectra obtained using the individual instruments. For green excitation instruments (532 nm) excellent spectra were obtained with strong Raman bands corresponding to the carotenoids. In this case spectral parameters were used directly as extracted from the spectra without any treatment. In the case of the 785 nm excitation baseline correction was necessary due to increased background and noise. The baseline was corrected manually using the GRAMS multipoint mode. The results coming from the Bravo PSSERS system are already treated in the frame of the fluorescence mitigation procedure, and the resulting spectra are therefore baseline corrected. The final spectrum is computed from six raw spectra.

## Results and Discussion

Carotenoids are commonly analyzed by Raman spectrometry using green excitation lasers close to 514 nm. Under such conditions, the presence of polyenic chains is the reason of the enhancement of the signal through the Resonance Raman effect (Merlin, 1985; Marshall et al., 2007). Raman spectroscopy has an excellent sensitivity for detecting polyenes, including carotenoids, because of the characteristic strong spectral signatures of the conjugated C–C and C=C functional modes. In particular this is enhanced by several orders of magnitude through resonance Raman excitation effects occurring between 480 and 532 nm laser wavelengths. Ideally, by reading the precise Raman shifts and mainly those of the C=C stretching band at around 1500–1540 cm^−1^ one can discriminate between carotenoids with different chain lengths. The precise position of the ν_1_ Raman feature (related to the C=C stretching vibrations) in the spectrum of a carotenoid depends mainly on the length of its polyenic chain. The longer the chain (the higher the number of the conjugated bonds), the lower the wavenumber at which the ν_1_ band occurs (Merlin, 1985; Withnall et al., 2003). However, for very similar carotenoids when the difference between the lengths of the polyenic chain is very small, the shifts are not enough to be used for unambiguous discrimination (de Oliveira et al., 2015; Malherbe et al., 2017). The interaction of carotenoid molecules with the hosting inorganic environment and the possible aggregation of several carotenoid molecules can also influence the band position (de Oliveira et al., 2010). Additionally, the spectral resolution and the stability of portable instrumentation do not allow very small shifts to correctly be recorded.

Here we recorded Raman signals of carotenoids in samples of cultures, using portable instruments. In two cases the used excitation radiation (785 nm and 785/853 nm) illuminating the targets was not close to the wavelength commonly used to enhance Raman signals through the resonance effect.

When focusing on carotenoids the best choice for excitation are green lasers. However, sometimes other lasers are used as well. Then, signals belonging to other compounds can also be obtained. For example, investigations of mineral and biomolecular characteristics of colonizations in travertines using a 785 nm diode laser allowed detecting the presence of scytonemin simultaneously with carotenoids and a suite of minerals (Jorge-Villar et al., 2007). Similarly, combining red and green lasers allows detecting pigments of organisms colonizing gypsum/halite crusts from hyperarid desert areas (Vítek et al., 2010). This was also made possible using two portable Raman spectrometers (Inspector Raman and First Guard) (Vítek et al., 2012).

The 1064 FT Raman modes in the microspectrometric arrangement (Holder et al., 2000; Fendrihan et al., 2009) can also be applied to study for instance geobiological samples. In this case the advantage can consist in the possibility to record Raman features of other compounds from the samples – both inorganic and organic (e.g., oxalate and carotenoids and/or additional pigment) without an enhancement of the polyenic chain signs.

**FIGURE 1:**
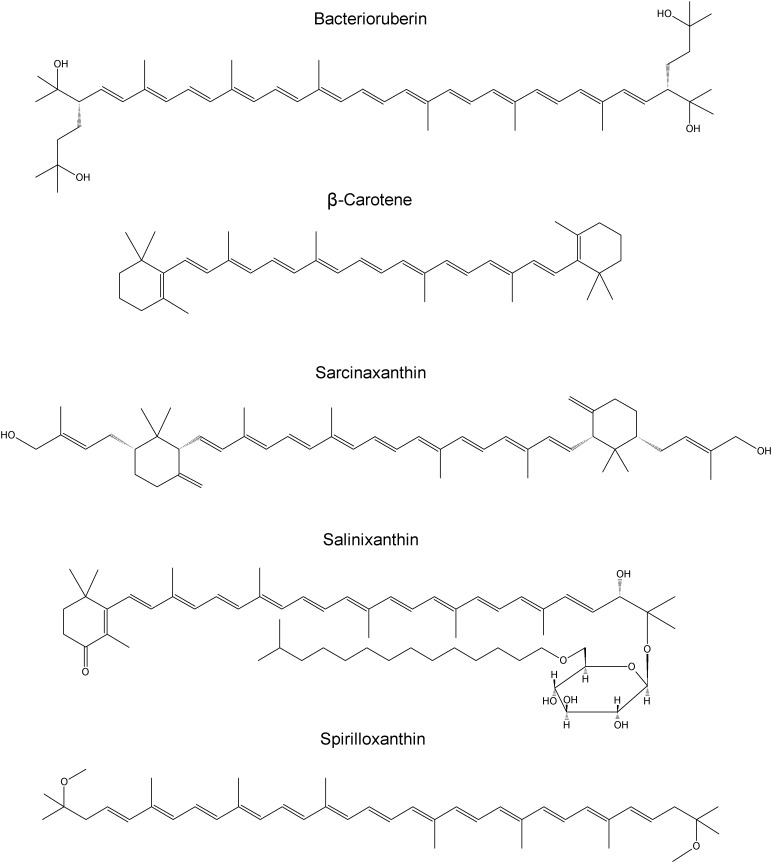
Structures of carotenoids of investigated microorganisms.

*Halobacterium salinarum*, *Halorubrum sodomense, Haloarcula marismortui* (as well as similar *Haloarcula vallismortis*) contain bacterioruberin, the main carotenoid component responsible for the characteristic color of the red Archaea of the class *Halobacteria*. The structure consists of a primary conjugated isoprenoid chain with 13 C=C units with no subsidiary conjugation arising from terminal groups, which contain four OH group functionalities. The observed Raman bands are expected at around 1506 cm^−1^ (C=C stretching band), 1152 cm^−1^ (stretching vibrations of C–C single bonds coupled with C–H in-plane bending modes) and 1001 cm^−1^ (in-plane rocking modes of CH_3_ groups attached to the polyene chain coupled with C–C bonds) (Marshall et al., 2007; Jehlička et al., 2013). For *Dunaliella parva* the characteristic carotenoid signatures lie at 1525, 1157, and 1005 cm^−1^ (Jehlička et al., 2014b). *Micrococcus luteus* is known to contain sarcinaxanthin with strong bands at 1528, 1158, and 1005 cm^−1^ (FT-Raman spectroscopy) assigned to C=C stretching, C–C stretching and C–CH_3_ deformation modes. In Raman spectra of *Ectothiorhodospira* and *Halochromatium*-like purple sulfur bacteria, especially spirilloxanthin-like carotenoids are expected to be detected. The main Raman bands recorded in *Ectothiorhodospira marismortui* are at 1514, 1152, and 1000 cm^−1^. Structures of carotenoids of investigated microorganisms are reported in Figure 1.

### Comparing the Instruments

**FIGURE 2:**
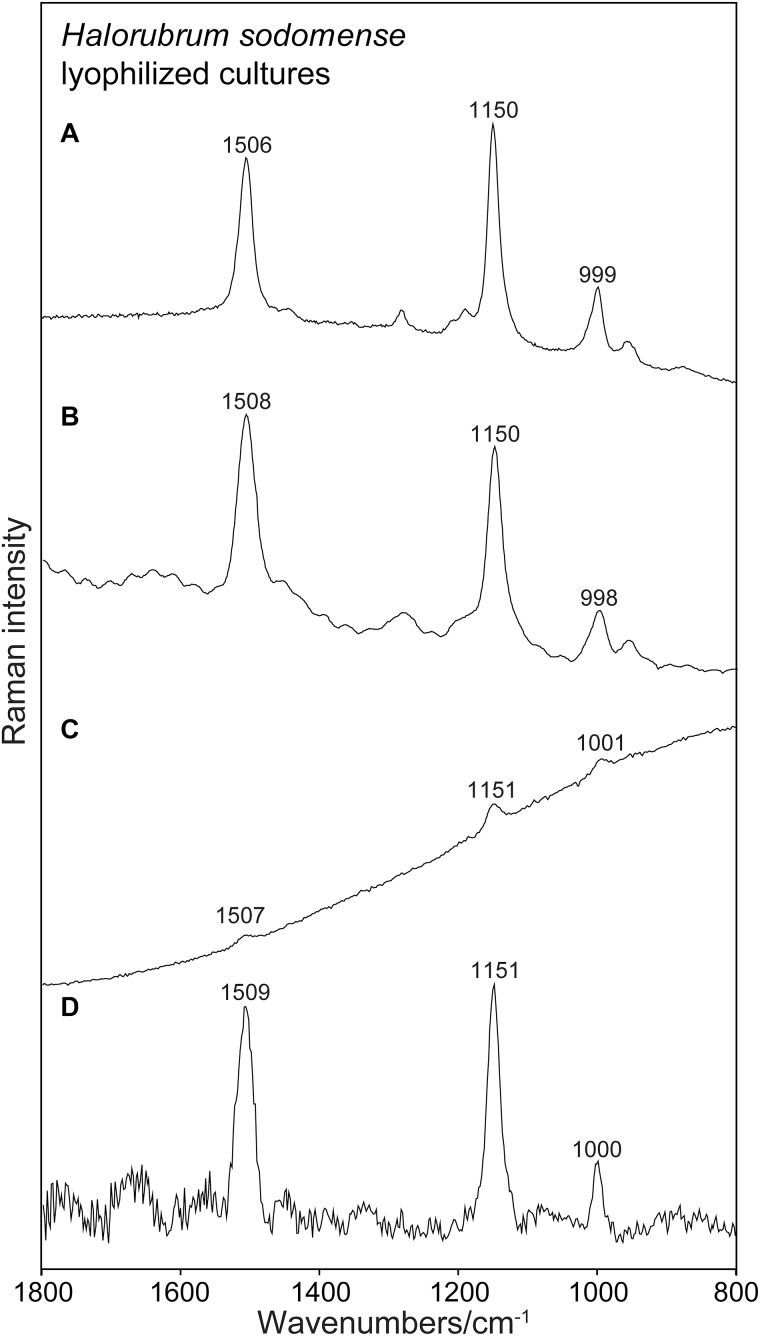
Raman spectra obtained for lyophilized cultures of *Halorubrum sodomense* using the four instruments. Spectra presented without any treatment. **(A)** RaPort, **(B)** First Guard, **(C)** Inspector Raman, **(D)** Bravo.

**FIGURE 3:**
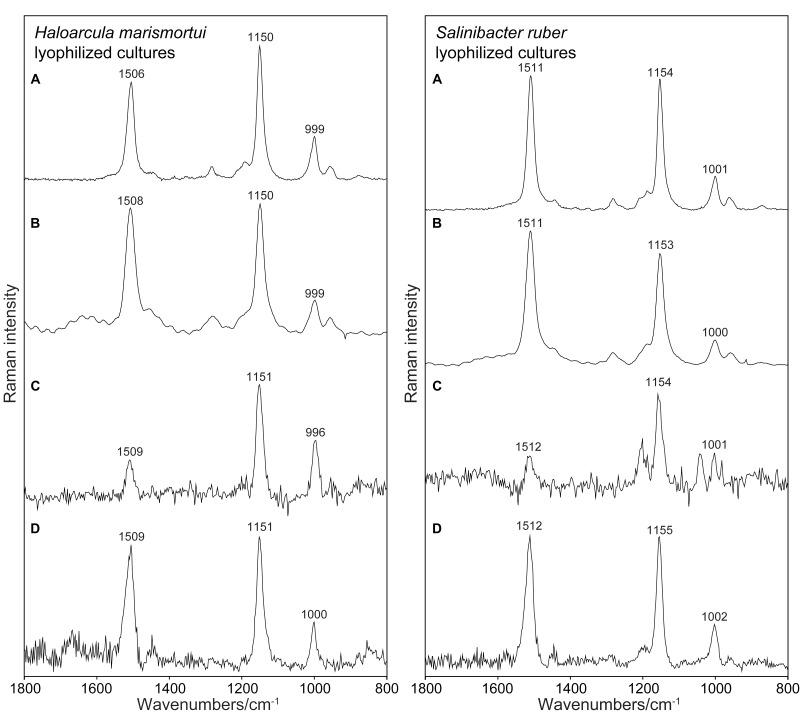
Raman spectra collected from lyophilized cultures of *Haloarcula marismortui* and *Salinibacter ruber* using the four instruments, baseline-corrected. **(A)** RaPort, **(B)** First Guard, **(C)** Inspector Raman, **(D)** Bravo.

All the instruments tested allowed to collect Raman spectra of carotenoids pigments. The general shape of the obtained spectra differs, and the raw spectra differ between instruments in their baseline characteristics. The strong signals of carotenoids are found at the correct positions known from microspectrometric reference measurements. Faint signals are collected in the case of the 785 nm excitation. Here spectra are collected under non-resonant conditions. However, subtracting the baseline allows reading of sharp and strong signals at correct positions also in this case. It is surprising that also the PSSRS allowed to obtain strong and correct Raman bands out of resonance. Raman spectrum that is recorded with the Bravo instrument and shown for instance in Figure 2D is also reported without any spectral manipulations in this illustration. The appearance of this spectrum with its flat baseline is a result of the fluorescence removal mechanism of this PSSER spectrometer. In Figure 3 the same flat spectrum is compared to the baseline corrected spectra taken with the other three instruments.

### Detection of Carotenoids Under Resonant and Non-resonant Conditions: 532 and 785 nm

Figure 2 presents an example of Raman spectra obtained using the four instruments without any data processing. Figure 2 shows examples of Raman spectra after baseline corrections. The common type of sample, lyophilized material that is used in general for spectroscopic investigation, was examined here. In all the cases strong Raman signals occurring at the correct positions are present in the spectra. No other signals occur in the spectra due to the prominent presence of carotenoids and due to the resonance enhancement of the signals of the conjugated bonds of the polyenic chain in the case of 532 nm spectra (Figure 2A,B). It is not surprising that the best results (correct wavenumbers, lower SD) were obtained using the green laser excitation with resonance Raman signals collected (Figure 2A,B). In these cases also faint signals were found, corresponding to the features commonly registered when using Raman microspectrometric laboratory devices.

It is interesting that Raman features of carotenoids are well visible and dominant also in the non-resonant mode obtained by the near infrared excitation or the PSSERS. This is especially true for the red colored halophiles, for which even the Inspector Raman instrument equipped with a common near infrared excitation was able to record good quality spectra of carotenoids. For the other microorganisms with higher concentration of different non-carotenoid pigments the 785 nm instrument consistently failed to record any usable spectra due to the excessive fluorescence. It was, however, possible to acquire a relatively strong signal of carotenoid pigment using the PSSERS on these samples (see Table 1). Therefore, for this kind of samples the combination of two excitation wavelengths and the fluorescence removal was definitely a great improvement for carotenoid pigment detection.

**Table 1:** An overview of all types of samples analyzed using different instruments.

Sample type	Culture type → Instrument ↓	*Halobacterium* R1	*Haloarcula marismortui*	*Halorubrum sodomense*	*Salinibacter ruber*	*Ectothiorhodo-spira marismortui*	*Dunaliella parva*	*Micrococcus luteus*	*Corynebacterium glutamicum*	Number of microorganisms for which carotenoid bands were detected out of 8 possible
Wet pellets	RaPort	✓	✓	✓	✓	✓	✓	✓	✓	8
Wet pellets	First Guard	✓	✓	✓	✓	✓	✓	✓	x	7
Wet pellets	Inspector Raman	✓	✓	✓	✓	x	x	x	x	4
Wet pellets	Bravo	✓	✓	✓	✓	x	✓	✓	x	6
Lyophilized cultures	RaPort	✓	✓	✓	✓	x	✓	✓	✓	7
Lyophilized cultures	First Guard	✓	✓	✓	✓	✓	✓	✓	✓	8
Lyophilized cultures	Inspector Raman	✓	✓	✓	✓	x	x	x	x	4
Lyophilized cultures	Bravo	✓	✓	✓	✓	x	✓	✓	✓	7
Methanol acetone extracts	RaPort	✓	✓	✓	✓	x	x	x	x	4
Methanol acetone extracts	First Guard	✓	✓	✓	✓	x	x	x	x	4
Methanol acetone extracts	Inspector Raman	✓	✓	✓	✓	x	x	x	x	4
Methanol acetone extracts	Bravo	✓	✓	✓	✓	x	✓	x	x	5
Bligh and Dyer extracts	RaPort	✓	✓	✓	✓	✓	x	✓	x	6
Bligh and Dyer extracts	First Guard	✓	✓	✓	✓	✓	x	✓	x	6
Bligh and Dyer extracts	Inspector Raman	✓	✓	✓	✓	x	x	x	x	4
Bligh and Dyer extracts	Bravo	✓	✓	✓	✓	x	✓	✓	✓	7

*A green OK sign signifies that with this specific combination of sample preparation and instrument used for analysis, the resulting Raman spectra contained carotenoid signals of satisfactory quality. Usually the three typical carotenoid bands were present. Red crosses denote that either no Raman signal was detected in the spectra, or that no spectra were acquired.*

### Comparing Sample Preparation

**FIGURE 4:**
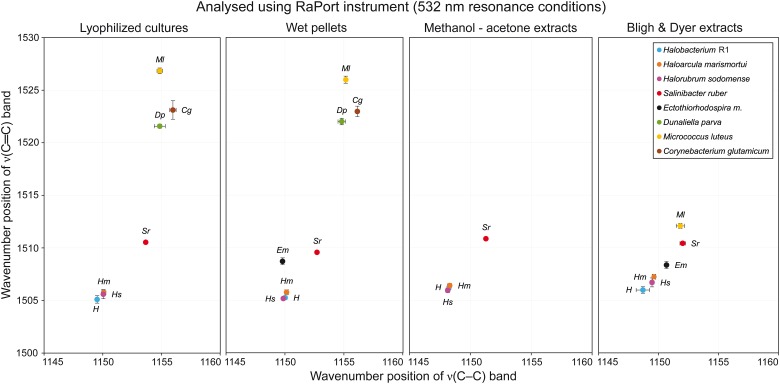
Graphs plotting wavenumber positions of ν(C–C) versus ν(C=C) Raman bands of carotenoids for all microorganisms tested in specified sample types under resonance conditions.

**FIGURE 5:**
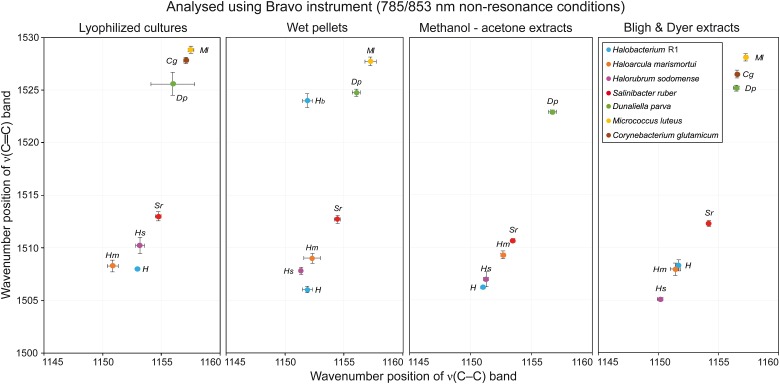
Graphs plotting wavenumber positions of ν(C–C) versus ν(C=C) of Raman bands of carotenoids for all microorganisms tested in specified sample types under out-of-resonance conditions.

Table 1 presents a general overview of all types of samples that were analyzed by different instruments. A green OK sign signifies that with this specific combination of sample preparation and instrument used for analysis, the resulting Raman spectra contained a carotenoid signal of satisfactory quality; usually the three typical carotenoid bands were present. Red crosses denote that either no Raman signal was detected in the spectra, or even that no spectra were acquired at all. The most typical problem encountered was fluorescence caused by other constituents in the samples. On the other hand, in the samples where the concentration of carotenoids is generally higher, typically in samples derived from the halophilic organisms, good Raman signals of carotenoids were recorded for all sample type preparations and all instruments. When comparing the different types of sample preparations it is evident that they can be arranged in descending order based on the number of successful detections of carotenoid pigments as follows: lyophilized cultures, wet pellets, Bligh and Dyer extracts, methanol-acetone extracts. The differences in the Raman spectra obtained when four different sample preparations were analyzed are discussed below, based on the Raman spectra taken with the RaPort instrument, and separately with the Bravo instrument for Raman spectra acquired under out-of-resonance conditions. Graphs plotting ν(C–C) versus ν(C=C) wavenumber positions for each microorganism in specified sample types are given in Figure 4 (resonance conditions) and Figure 5 (out-of-resonance conditions).

### Resonance Conditions

#### Wet Pellets

Very similar results were obtained when the first four microorganisms prepared from wet pellets were analyzed. The ν_1_(C=C) band was located at 1505–1506 cm^−1^ for *Hbt. salinarum*, *Har. marismortui* and *Hrr. sodomense* and at 1509–1510 cm^−1^ for *S. ruber*. For this type of samples very good quality spectra were obtained for *E. marismortui* with the ν_1_(C=C) band position at 1508–1509 cm^−1^. The Raman spectra obtained for the last three microorganisms, similarly, exhibited a high intensity fluorescence, but the positions of the ν_1_(C=C) band that were obtained for *D. parva*, *M. luteus*, and *C. glutamicum* were at around 1522, 1526, and 1523 cm^−1^, respectively, so very similar when compared to the lyophilized cultures sample.

#### Lyophilized Cultures

For *Hbt. salinarum*, *Har. marismortui*, and *Hrr. sodomense* (Archaea) the ν_1_(C=C) band is located at 1505–1506 cm^−1^. For *S. ruber* (Bacteria), this band is located at 1510–1511 cm^−1^. Raman spectra of these halophilic organisms prepared as lyophilized cultures exhibit strong signals of carotenoids and only relatively low fluorescence background. For *E. marismortui* it was impossible to acquire Raman spectra due to extensive fluorescence. For the green alga *D. parva* the ν_1_(C=C) band is located at 1521–1522 cm^−1^, and the spectra typically exhibit intense fluorescence. Therefore, the baseline correction procedure was used here. Raman spectra of *M. luteus* also exhibited moderately high fluorescence, necessitating baseline correction, and the ν_1_(C=C) band is located at 1526–1527 cm^−1^. Finally, for *C. glutamicum* an intense fluorescence background was observed, but the Raman signal of carotenoids was clearly present, the ν_1_(C=C) band being located at around 1523 cm^−1^.

#### Methanol-Acetone Extracts

With this type of samples it was possible to record good quality data only for *Hbt. salinarum*, *Har. marismortui*, *Hrr. sodomense*, and *S. ruber*. Compared to the first two sample types, the Raman spectra exhibited increased noise, however, the characteristic carotenoid Raman bands were still of moderate to high intensity. The wavenumber positions of characteristic bands were again very similar to those in the first two types of samples. For *Hbt. salinarum*, *Har. marismortui*, and *Hrr. sodomense* the ν_1_(C=C) band is located at around 1506 cm^−1^ and for *S. ruber* at around 1511 cm^−1^. Raman spectra of *E. marismortui*, *M. luteus*, and *C. glutamicum* typically contained only high fluorescence background with occasional bands due to the solvents. It was impossible to record spectra of *D. parva* due to the detector saturation, probably due to excessive fluorescence. This result reflects the low extraction efficiency through methanol-acetone extraction. A better approach consists in the well established Bligh and Dyer procedure of extracting carotenoids.

#### Bligh and Dyer Extracts

This type of samples provided good quality Raman spectra with slightly higher noise than for the first two sample types. For *Hbt. salinarum*, *Har. marismortui*, and *Hrr. sodomense* the ν_1_(C=C) band is located at 1506–1507 cm^−1^. For *S. ruber*, this band is located at around 1510 cm^−1^. Raman spectra of *E. marismortui* exhibited very high fluorescence, however, it was possible to obtain the ν_1_(C=C) band positions at around 1508 cm^−1^ after baseline correction. Again, it was impossible to record spectra of *D. parva* due to the detector saturation. Very small intensity bands were located at around 1522 cm^−1^ for *C. glutamicum*, but no other carotenoid bands were found. The most interesting results came from analyses of B&D extracts of *M. luteus*. In this type of samples, the three main carotenoid bands were detected at positions around 1001, 1152, and 1512 cm^−1^. This is a significant shift (especially for the ν_1_(C=C) band) from the values taken on the “non-extracted” samples: 1004–1004, 1155, and 1526–1527 cm^−1^. The plausible explanation for such a great shift in wavenumbers is that the carotenoid composition in the extract differs from that in the original culture.

### Out-of-Resonance Conditions

Here we present the results obtained using the portable Raman spectrometer equipped with dual sequentially-shifted 785/853 nm excitation. Raman spectra coming from this instrument are baseline corrected by design as unwanted fluorescence is removed. On the other hand, this instrument uses lasers that do not meet the resonance condition for carotenoid molecules, and therefore the carotenoid signal in the Raman spectra should be lower. Indeed the spectra appear much noisier, in several cases instrumental artifact bands appear in the spectra. However, it is generally possible to obtain wavenumber values for the three most significant carotenoid bands.

#### Wet Pellets

Two distinct ν_1_(C=C) Raman bands with a good separation in wavenumbers were detected in the spectra of *Hbt. salinarum*. Precise values were obtained using a peak-fitting procedure, with the following outcome: a slightly more intense band located at around 1524 cm^−1^ and a second band located at approximately 1506 cm^−1^. For *Har. marismortui*, and *Hrr. sodomense* the average position of the ν_1_(C=C) Raman band was 1509 and 1508 cm^−1^, respectively. The best carotenoid signal was recorded for *S. ruber* with the band located at around 1513 cm^−1^. For *E. marismortui* it was impossible to acquire Raman spectra due to extensive fluorescence and related instrumental artifacts. A rather intense signal, even under the out-of-resonance conditions, was recorded for *D. parva*, where the ν_1_(C=C) band is located at around 1525 cm^−1^. Raman spectra of *M. luteus* were of good quality, with this band located at approximately 1528 cm^−1^. No Raman bands of any kind were detected in the very noisy spectra for *C. glutamicum*.

#### Lyophilized Cultures

For *Hbt. salinarum* and *Har. marismortui* the ν_1_(C=C) band is located at around 1508 cm^−1^. In the spectra of *Hrr. sodomense* this band is located at around 1510 cm^−1^. For *S. ruber*, this band can be found at 1513 cm^−1^. The intensity of the carotenoid bands is highest for spectra of *S*. *ruber*, and lowest for the spectra of *H. salinarum*. For *E. marismortui* it was impossible to acquire Raman spectra due to extensive fluorescence which in this case manifests as a very high intensity instrumental artifacts bands in the final sequentially shifted spectra. For *D. parva* the ν_1_(C=C) band is located at 1525–1526 cm^−1^ in the spectra typically exhibiting intense artifact background. Raman spectra of *M. luteus* were of good quality and the ν_1_(C=C) band is located at around 1529 cm^−1^. For *C. glutamicum* a moderate carotenoid signal was present with the ν_1_(C=C) band located at around 1528 cm^−1^.

#### Methanol-Acetone Extracts

Clear carotenoid signals with a good signal-to-noise ratio were obtained for the red to purple colored microorganisms. The position of the most important Raman band, the ν_1_(C=C) band, was recorded at approximately 1506 cm^−1^ for *Hbt. salinarum*. A slight upshift in wavenumbers compared with the resonance conditions was detected for *Har. marismortui* and *Hrr. sodomense*, corresponding wavenumber values being 1509 and 1507 cm^−1^. For *S. ruber*, the ν_1_(C=C) band position is at 1511 cm^−1^. For *E. marismortui* only instrumental artifacts were found in the spectra. Good quality Raman spectra were recorded for *D. parva*, the wavenumber value for the most intense band being 1523 cm^−1^. No usable Raman bands were found in the spectra for *M. luteus*, and *C. glutamicum* typically yielded intense noise background or instrumental artifact bands.

#### Bligh and Dyer Extracts

For *Hbt. salinarum* and *Har. marismortui* the ν_1_(C=C) band is located at around 1508 cm^−1^, the same value as in spectra of lyophilized cultures samples. However, the intensity of the carotenoid signal is significantly higher for B&D extracts. In the spectra of *Hrr. sodomense* this band is located at around 1505 cm^−1^. This is a significant downshift from the values around 1510 cm^−1^ for the lyophilized cultures samples. For *S. ruber*, the ν_1_(C=C) band is found at around 1512 cm^−1^, a value similar to that found for the lyophilized cultures samples. No usable Raman spectra of *E. marismortui* were acquired for similar reasons as mentioned previously. For *D. parva* the ν_1_(C=C) band is located at around 1525 cm^−1^, again a comparable value. The same is true for Raman spectra of *M. luteus* where the ν_1_(C=C) band is located at around 1528 cm^−1^. For *C. glutamicum* a good quality carotenoid signal was present with the ν_1_(C=C) band located at around 1527 cm^−1^.

Interesting results come from analyses of B&D extracts of *M. luteus*. In this type of samples, the three main carotenoid bands were detected at positions around 1001, 1152, and 1512 cm^−1^. This is a significant shift (especially for the ν_1_(C=C) band) from the values taken on the “non-extracted” samples: 1004–1004, 1155, and 1526–1527 cm^−1^, respectively. The plausible explanation for such a great shift in wavenumbers is that during the extraction the structure and composition of the carotenoid was modified compared to the bound state of the pigment in the initial culture of *M. luteus*.

## Conclusion

From the point of view of detection of Raman bands of carotenoid pigments, the two sample preparations that were found the best are lyophilized cultures and wet pellets (see Supplementary Tables). This means that the highest number of Raman bands of carotenoids, usually with good signal-to-noise ratio, is detected in these types of sample preparations. Bligh and Dyer extracts also provided a good detection for the carotenoid bands. Some changes in the position of Raman bands of carotenoids (probably due to the change in carotenoid composition during the extraction process) were encountered for the *M. luteus* samples. The methanol-acetone extracts offered the worst detection of the carotenoid Raman bands, with additional Raman bands due to the solvents, and usually only the red/purple colored microorganisms provided Raman spectra of usable quality for carotenoid detection.

Compact lightweight portable Raman spectrometers equipped with 514, 785, and dual 785/853 nm excitation lasers were used to detect pigments in a series of halophilic (Archaea of the class *Halobacteria*, *Salinibacter*) and non-halophilic microorganisms (*Micrococcus luteus*, *Corynebacterium glutamicum*). Common as well as less common carotenoids, including α-bacterioruberin, salinixanthin and spirilloxanthin derivatives, were detected in culture samples of model organisms belonging to the genera *Halobacterium*, *Haloarcula*, *Halorubrum* (Archaea),*Salinibacter* (*Bacteroidetes*), *Ectothiorhodospira* (*Gammaproteobacteria*) *Dunaliella* (*Chlorophyceae*), *Micrococcus*, and *Corynebacterium* (*Actinobacteria*).

Excellent reliability and robustness of collected major diagnostic band positions were confirmed in the case of the RaPort portable system. Interestingly, very good correspondence of values obtained by a PSSERS operating with lasers not allowing collecting resonance Raman signals of carotenoid chains was observed.

## Data Availability

The datasets generated for this study are available on request to the corresponding author.

## Author Contributions

JJ and AO conceived and designed the project. AO and LM prepared the cultures and samples. AC and JJ carried out the experiments. JJ, AC, and AO wrote the manuscript.

## Conflict of Interest Statement

The authors declare that the research was conducted in the absence of any commercial or financial relationships that could be construed as a potential conflict of interest.
