# Supplementary material for: Comparison of Miniaturized Raman Spectrometers for Discrimination of Carotenoids of Halophilic Microorganisms
Source: Front Microbiol. 2019 May 29;10:1155. doi: 10.3389/fmicb.2019.01155 (PMC6548819; doi:10.3389/fmicb.2019.01155)
Supplement: Supplementary file 1 [file Table_1.docx]

***Supplementary Material***

**1 Supplemetary Tables**

Tables listing wavenumber positions (cm^-1^) as well as standard deviations (each band position was calculated as an average of five measurements) of the three characteristic Raman bands of detected carotenoid pigments. The tables contain all the data, as was collected using four instruments analysing eight microorganisms, each of them prepared in four different types of samples. An x denotes a band was not detected.

| ***Halobacterium* *salinarum*** | **ρ(C–CH_3_)** | **sd1** | **ν_2_(C–C)** | **sd2** | **ν_1_(C=C)** | **sd3** |
| --- | --- | --- | --- | --- | --- | --- |
| Wet pellets | 999 | 0.07 | 1150 | 0.16 | 1505.2 | 0.08 |
| Lyophilized cells | 999 | 0.19 | 1149.5 | 0.1 | 1505.1 | 0.35 |
| Methanol-acetone extracts | 1000.0 | 0.1 | 1148.3 | 0.1 | 1506.3 | 0.3 |
| Bligh & Dyer extracts | 999.7 | 0.43 | 1148.7 | 0.57 | 1506.0 | 0.31 |

| ***Haloarcula marismortui*** | **ρ(C–CH_3_)** | **sd1** | **ν_2_(C–C)** | **sd2** | **ν_1_(C=C)** | **sd3** |
| --- | --- | --- | --- | --- | --- | --- |
| Wet pellets | 1000.1 | 0.17 | 1150.1 | 0.08 | 1505.8 | 0.23 |
| Lyophilized cells | 999.4 | 0.19 | 1149.5 | 0.1 | 1505.1 | 0.35 |
| Methanol-acetone extracts | 1000.1 | 0.5 | 1148.3 | 0.1 | 1506.4 | 0.2 |
| Bligh & Dyer extracts | 999.8 | 0.26 | 1149.6 | 0.15 | 1507.2 | 0.22 |

| ***Halorubrum sodomense*** | **ρ(C–CH_3_)** | **sd1** | **ν_2_(C–C)** | **sd2** | **ν_1_(C=C)** | **sd3** |
| --- | --- | --- | --- | --- | --- | --- |
| Wet pellets | 999.1 | 0.14 | 1149.9 | 0.08 | 1505.2 | 0.14 |
| Lyophilized cells | 1000 | 1.1 | 1150.5 | 0.99 | 1506.1 | 1.07 |
| Methanol-acetone extracts | 999.7 | 0.2 | 1148.1 | 0.1 | 1505.9 | 0.2 |
| Bligh & Dyer extracts | 1000 | 0.38 | 1149.5 | 0.16 | 1506.7 | 0.42 |

| ***Salinibacter ruber*** | **ρ(C–CH_3_)** | **sd1** | **ν_2_(C–C)** | **sd2** | **ν_1_(C=C)** | **sd3** |
| --- | --- | --- | --- | --- | --- | --- |
| Wet pellets | 1000.8 | 0.3 | 1152.7 | 0.07 | 1509.6 | 0.14 |
| Lyophilized cells | 1001.0 | 0.27 | 1153.7 | 0.14 | 1510.5 | 0.12 |
| Methanol-acetone extracts | 1002.6 | 0.7 | 1151.3 | 0.1 | 1510.9 | 0.1 |
| Bligh & Dyer extracts | 1000.8 | 0.30 | 1152.0 | 0.23 | 1510.4 | 0.15 |

| ***Ectothiorhodosphira marismortui*** | **ρ(C–CH_3_)** | **sd1** | **ν_2_(C–C)** | **sd2** | **ν_1_(C=C)** | **sd3** |
| --- | --- | --- | --- | --- | --- | --- |
| Wet pellets | 1001.2 | 0.15 | 1149.8 | 0.09 | 1508.7 | 0.33 |
| Lyophilized cells | x | x | x | x | x | x |
| Methanol-acetone extracts | x | x | x | x | x | x |
| Bligh & Dyer extracts | 1000.7 | 0.13 | 1150.7 | 0.26 | 1508.4 | 0.14 |

| ***Dunaliella parva*** | **ρ(C–CH_3_)** | **sd1** | **ν_2_(C–C)** | **sd2** | **ν_1_(C=C)** | **sd3** |
| --- | --- | --- | --- | --- | --- | --- |
| Wet pellets | 1004.1 | 1.02 | 1154.8 | 0.32 | 1522 | 0.32 |
| Lyophilized cells | 1004.4 | 1.3 | 1154.9 | 0.47 | 1521.6 | 0.19 |
| Methanol-acetone extracts | x | x | x | x | x | x |
| Bligh & Dyer extracts | x | x | x | x | x | x |

| ***Micrococcus luteus*** | **ρ(C–CH_3_)** | **sd1** | **ν_2_(C–C)** | **sd2** | **ν_1_(C=C)** | **sd3** |
| --- | --- | --- | --- | --- | --- | --- |
| Wet pellets | 1003.6 | 0.7 | 1155.2 | 0.12 | 1526 | 0.34 |
| Lyophilized cells | 1005.3 | 0.8 | 1154.9 | 0.24 | 1526.8 | 0.27 |
| Methanol-acetone extracts | x | x | x | x | x | x |
| Bligh & Dyer extracts | 1001.3 | 0.13 | 1151.8 | 0.34 | 1512.1 | 0.25 |

| ***Corynebacterium glutamicum*** | **ρ(C–CH_3_)** | **sd1** | **ν_2_(C–C)** | **sd2** | **ν_1_(C=C)** | **sd3** |
| --- | --- | --- | --- | --- | --- | --- |
| Wet pellets | 1004.8 | 0.38 | 1156.1 | 0.15 | 1523 | 0.5 |
| Lyophilized cells | 1006.3 | 0.59 | 1156 | 0.27 | 1523.1 | 0.88 |
| Methanol-acetone extracts | x | x | x | x | x | x |
| Bligh & Dyer extracts | x | x | x | x | 1522? | x |

**Tables for RaPort (532 nm)**

| ***Halobacterium* *salinarum*** | **ρ(C–CH_3_)** | **sd1** | **ν_2_(C–C)** | **sd2** | **ν_1_(C=C)** | **sd3** |
| --- | --- | --- | --- | --- | --- | --- |
| Wet pellets | 998.0 | 0.23 | 1149.7 | 0.05 | 1506.2 | 0.07 |
| Lyophilized cells | 999.0 | 0.77 | 1150.3 | 0.04 | 1507.5 | 0.3 |
| Methanol-acetone extracts | 998.7 | 0.7 | 1147.4 | 0.1 | 1506.5 | 0.2 |
| Bligh & Dyer extracts | 998.4 | 0.48 | 1148.7 | 0.05 | 1507.0 | 0.13 |

| ***Haloarcula marismortui*** | **ρ(C–CH_3_)** | **sd1** | **ν_2_(C–C)** | **sd2** | **ν_1_(C=C)** | **sd3** |
| --- | --- | --- | --- | --- | --- | --- |
| Wet pellets | 998.8 | 0.58 | 1149.7 | 0.05 | 1506.4 | 0.05 |
| Lyophilized cells | 998.4 | 0.56 | 1149.8 | 0.05 | 1508.0 | 0.13 |
| Methanol-acetone extracts | 999.0 | 0.3 | 1147.2 | 0.0 | 1506.4 | 0.3 |
| Bligh & Dyer extracts | 998.6 | 0.05 | 1148.7 | 0.04 | 1507.0 | 0.07 |

| ***Halorubrum sodomense*** | **ρ(C–CH_3_)** | **sd1** | **ν_2_(C–C)** | **sd2** | **ν_1_(C=C)** | **sd3** |
| --- | --- | --- | --- | --- | --- | --- |
| Wet pellets | 997.8 | 0.15 | 1149.7 | 0.00 | 1506.2 | 0.04 |
| Lyophilized cells | 999.3 | 0.35 | 1149.9 | 0.05 | 1507.4 | 0.27 |
| Methanol-acetone extracts | 999.7 | 0.2 | 1148.8 | 0.1 | 1507.4 | 0.1 |
| Bligh & Dyer extracts | 998.2 | 0.42 | 1148.6 | 0.00 | 1506.9 | 0.05 |

| ***Salinibacter ruber*** | **ρ(C–CH_3_)** | **sd1** | **ν_2_(C–C)** | **sd2** | **ν_1_(C=C)** | **sd3** |
| --- | --- | --- | --- | --- | --- | --- |
| Wet pellets | 1000.3 | 0.12 | 1152.3 | 0.06 | 1510.3 | 0.07 |
| Lyophilized cells | 1001.0 | 0.78 | 1152.5 | 0.05 | 1510.6 | 0.04 |
| Methanol-acetone extracts | 1002.0 | 0.3 | 1151.3 | 0.2 | 1510.9 | 0.4 |
| Bligh & Dyer extracts | 1000.3 | 0.27 | 1151.5 | 0.08 | 1509.7 | 0.05 |

| ***Ectothiorhodosphira marismortui*** | **ρ(C–CH_3_)** | **sd1** | **ν_2_(C–C)** | **sd2** | **ν_1_(C=C)** | **sd3** |
| --- | --- | --- | --- | --- | --- | --- |
| Wet pellets | 999.8 | 0.1 | 1149.4 | 0.32 | 1508.7 | 0.19 |
| Lyophilized cells | 997.8 | 1.13 | 1149.8 | 0.1 | 1508.6 | 0.43 |
| Methanol-acetone extracts | x | x | x | x | x | x |
| Bligh & Dyer extracts | 997.6 | 0.46 | 1149.5 | 0.08 | 1507.8 | 0.39 |

| ***Dunaliella parva*** | **ρ(C–CH_3_)** | **sd1** | **ν_2_(C–C)** | **sd2** | **ν_1_(C=C)** | **sd3** |
| --- | --- | --- | --- | --- | --- | --- |
| Wet pellets | 1000.9 | 1.06 | 1153.3 | 0.14 | 1520.4 | 0.47 |
| Lyophilized cells | 1001.5 | 0.91 | 1152.8 | 0.48 | 1519.8 | 0.79 |
| Methanol-acetone extracts | x | x | x | x | x | x |
| Bligh & Dyer extracts | x | x | x | x | x | x |

| ***Micrococcus luteus*** | **ρ(C–CH_3_)** | **sd1** | **ν_2_(C–C)** | **sd2** | **ν_1_(C=C)** | **sd3** |
| --- | --- | --- | --- | --- | --- | --- |
| Wet pellets | 1000.4 | 0.00 | 1156.1 | 0.43 | 1524.8 | 1.83 |
| Lyophilized cells | 1001.1 | 1.55 | 1154.0 | 0.73 | 1522.8 | 0.56 |
| Methanol-acetone extracts | x | x | x | x | x | x |
| Bligh & Dyer extracts | 1001.2 | 0.74 | 1151.1 | 0.58 | 1510.3 | 0.62 |

| ***Corynebacterium glutamicum*** | **ρ(C–CH_3_)** | **sd1** | **ν_2_(C–C)** | **sd2** | **ν_1_(C=C)** | **sd3** |
| --- | --- | --- | --- | --- | --- | --- |
| Wet pellets | x | x | x | x | x | x |
| Lyophilized cells | – | – | 1153.1 | 0.25 | 1521.6 | 0.59 |
| Methanol-acetone extracts | x | x | x | x | x | x |
| Bligh & Dyer extracts | x | x | x | x | x | x |

**Tables for First Guard (532 nm)**

| ***Halobacterium* *salinarum*** | **ρ(C–CH_3_)** | **sd1** | **ν_2_(C–C)** | **sd2** | **ν_1_(C=C)** | **sd3** |
| --- | --- | --- | --- | --- | --- | --- |
| Wet pellets | 998.7 | 0.34 | 1149.4 | 0.21 | 1506.6 | 0.48 |
| Lyophilized cells | 1000.2 | 0.86 | 1151.5 | 0.12 | 1508.5 | 0.63 |
| Methanol-acetone extracts | – | – | 1150.6 | 0,3 | 1506,3 | 0,37 |
| Bligh & Dyer extracts | – | – | 1149.7 | 0.46 | 1508.7 | 0.38 |

| ***Haloarcula marismortui*** | **ρ(C–CH_3_)** | **sd1** | **ν_2_(C–C)** | **sd2** | **ν_1_(C=C)** | **sd3** |
| --- | --- | --- | --- | --- | --- | --- |
| Wet pellets | 998.0 | 0.25 | 1149.2 | 0.08 | 1506.2 | 0.32 |
| Lyophilized cells | 999.7 | 2.04 | 1151.1 | 0.33 | 1507.8 | 0.52 |
| Methanol-acetone extracts | – | – | 1149,2 | 0,08 | 1506,6 | 0,1 |
| Bligh & Dyer extracts | – | – | 1149.5 | 0.53 | 1510.2 | 0.52 |

| ***Halorubrum sodomense*** | **ρ(C–CH_3_)** | **sd1** | **ν_2_(C–C)** | **sd2** | **ν_1_(C=C)** | **sd3** |
| --- | --- | --- | --- | --- | --- | --- |
| Wet pellets | 997.9 | 0.22 | 1149.3 | 0.07 | 1505.8 | 0.19 |
| Lyophilized cells | 1000.6 | 0.44 | 1151.5 | 0.05 | 1508.0 | 0.64 |
| Methanol-acetone extracts | – | – | 1149,1 | 0,23 | 1502,2 | 0,43 |
| Bligh & Dyer extracts | 1001.3 | 0.44 | 1149.1 | 0.05 | 1508.7 | 0.37 |

| ***Salinibacter ruber*** | **ρ(C–CH_3_)** | **sd1** | **ν_2_(C–C)** | **sd2** | **ν_1_(C=C)** | **sd3** |
| --- | --- | --- | --- | --- | --- | --- |
| Wet pellets | 1000.7 | 0.19 | 1153.8 | 0.07 | 1512.2 | 0.15 |
| Lyophilized cells | 1001.1 | 0.28 | 1154.6 | 0.44 | 1512.5 | 0.12 |
| Methanol-acetone extracts | – | – | 1150,6 | 0,06 | 1507,9 | 0,1 |
| Bligh & Dyer extracts | – | – | 1152.5 | 0.52 | 1512.5 | 0.17 |

| ***Ectothiorhodosphira marismortui*** | **ρ(C–CH_3_)** | **sd1** | **ν_2_(C–C)** | **sd2** | **ν_1_(C=C)** | **sd3** |
| --- | --- | --- | --- | --- | --- | --- |
| Wet pellets | x | x | x | x | x | x |
| Lyophilized cells | x | x | x | x | x | x |
| Methanol-acetone extracts | x | x | x | x | x | x |
| Bligh & Dyer extracts | x | x | x | x | x | x |

| ***Dunaliella parva*** | **ρ(C–CH_3_)** | **sd1** | **ν_2_(C–C)** | **sd2** | **ν_1_(C=C)** | **sd3** |
| --- | --- | --- | --- | --- | --- | --- |
| Wet pellets | x | x | x | x | x | x |
| Lyophilized cells | x | x | x | x | x | x |
| Methanol-acetone extracts | x | x | x | x | x | x |
| Bligh & Dyer extracts | x | x | x | x | x | x |

| ***Micrococcus luteus*** | **ρ(C–CH_3_)** | **sd1** | **ν_2_(C–C)** | **sd2** | **ν_1_(C=C)** | **sd3** |
| --- | --- | --- | --- | --- | --- | --- |
| Wet pellets | x | x | x | x | x | x |
| Lyophilized cells | x | x | x | x | x | x |
| Methanol-acetone extracts | x | x | x | x | x | x |
| Bligh & Dyer extracts | x | x | x | x | x | x |

| ***Corynebacterium glutamicum*** | **ρ(C–CH_3_)** | **sd1** | **ν_2_(C–C)** | **sd2** | **ν_1_(C=C)** | **sd3** |
| --- | --- | --- | --- | --- | --- | --- |
| Wet pellets | x | x | x | x | x | x |
| Lyophilized cells | x | x | x | x | x | x |
| Methanol-acetone extracts | x | x | x | x | x | x |
| Bligh & Dyer extracts | x | x | x | x | x | x |

**Tables for Inspector Raman (785 nm)**

| ***Halobacterium* *salinarum*** | **ρ(C–CH_3_)** | **sd1** | **ν_2_(C–C)** | **sd2** | **ν_1_(C=C)** | **sd3** |
| --- | --- | --- | --- | --- | --- | --- |
| Wet pellets | 1002.8 | 0.64 | 1151.9 | 0.43 | 1506 | 0.30 |
| Lyophilized cells | 1002 | 0 | 1153 | 0 | 1508 | 0 |
| Methanol-acetone extracts | 1000.3 | 0.44 | 1151.0 | 0.08 | 1506.2 | 0.19 |
| Bligh & Dyer extracts | 1001.4 | 0.68 | 1151.7 | 0.17 | 1508.3 | 0.54 |

| ***Haloarcula marismortui*** | **ρ(C–CH_3_)** | **sd1** | **ν_2_(C–C)** | **sd2** | **ν_1_(C=C)** | **sd3** |
| --- | --- | --- | --- | --- | --- | --- |
| Wet pellets | 1002.2 | 0.95 | 1152.3 | 0.71 | 1509.0 | 0.47 |
| Lyophilized cells | 1001.2 | 0.44 | 1150.9 | 0.45 | 1508.3 | 0.55 |
| Methanol-acetone extracts | 1002.5 | 0.48 | 1152.7 | 0.19 | 1509.3 | 0.36 |
| Bligh & Dyer extracts | 1001.2 | 0.68 | 1151.4 | 0.41 | 1508 | 0.59 |

| ***Halorubrum sodomense*** | **ρ(C–CH_3_)** | **sd1** | **ν_2_(C–C)** | **sd2** | **ν_1_(C=C)** | **sd3** |
| --- | --- | --- | --- | --- | --- | --- |
| Wet pellets | 1000.9 | 0.33 | 1151.4 | 0.16 | 1507.8 | 0.32 |
| Lyophilized cells | 1002.6 | 0.29 | 1153.2 | 0.38 | 1510.2 | 0.77 |
| Methanol-acetone extracts | 1001.2 | 0.39 | 1151.3 | 0.22 | 1507.0 | 0.72 |
| Bligh & Dyer extracts | 1003 | 0.16 | 1150.2 | 0.24 | 1505.1 | 0.09 |

| ***Salinibacter ruber*** | **ρ(C–CH_3_)** | **sd1** | **ν_2_(C–C)** | **sd2** | **ν_1_(C=C)** | **sd3** |
| --- | --- | --- | --- | --- | --- | --- |
| Wet pellets | 1002.6 | 0.59 | 1154.5 | 0.25 | 1512.7 | 0.37 |
| Lyophilized cells | 1001.9 | 0.38 | 1154.8 | 0.27 | 1513 | 0.42 |
| Methanol-acetone extracts | 1001.3 | 0.58 | 1153.5 | 0.06 | 1510.7 | 0.21 |
| Bligh & Dyer extracts | 1001.7 | 0.41 | 1154.2 | 0.13 | 1512.3 | 0.30 |

| ***Ectothiorhodosphira marismortui*** | **ρ(C–CH_3_)** | **sd1** | **ν_2_(C–C)** | **sd2** | **ν_1_(C=C)** | **sd3** |
| --- | --- | --- | --- | --- | --- | --- |
| Wet pellets | x | x | x | x | x | x |
| Lyophilized cells | x | x | x | x | x | x |
| Methanol-acetone extracts | x | x | x | x | x | x |
| Bligh & Dyer extracts | x | x | x | x | x | x |

| ***Dunaliella parva*** | **ρ(C–CH_3_)** | **sd1** | **ν_2_(C–C)** | **sd2** | **ν_1_(C=C)** | **sd3** |
| --- | --- | --- | --- | --- | --- | --- |
| Wet pellets | – | – | 1156.1 | 0.33 | 1524.7 | 0.34 |
| Lyophilized cells | 1001.2 | 0.2 | 1156 | 1.86 | 1525.6 | 1.08 |
| Methanol-acetone extracts | 1007.3 | 0.65 | 1156.7 | 0.31 | 1522.9 | 0.19 |
| Bligh & Dyer extracts | – | – | 1156.5 | 0.24 | 1525.2 | 0.3 |

| ***Micrococcus luteus*** | **ρ(C–CH_3_)** | **sd1** | **ν_2_(C–C)** | **sd2** | **ν_1_(C=C)** | **sd3** |
| --- | --- | --- | --- | --- | --- | --- |
| Wet pellets | 1004.0 | 0.84 | 1157.3 | 0.49 | 1527.7 | 0.42 |
| Lyophilized cells | 1004.9 | 1.2 | 1157.5 | 0.24 | 1528.8 | 0.35 |
| Methanol-acetone extracts | x | x | x | x | x | x |
| Bligh & Dyer extracts | 1005.6 | 0.46 | 1157.3 | 0.18 | 1528.1 | 0.35 |

| ***Corynebacterium glutamicum*** | **ρ(C–CH_3_)** | **sd1** | **ν_2_(C–C)** | **sd2** | **ν_1_(C=C)** | **sd3** |
| --- | --- | --- | --- | --- | --- | --- |
| Wet pellets | x | x | x | x | x | x |
| Lyophilized cells | 1003.4 | 0.77 | 1157.1 | 0.19 | 1527.8 | 0.33 |
| Methanol-acetone extracts | x | x | x | x | x | x |
| Bligh & Dyer extracts | 1005.6 | 0.74 | 1156.6 | 0.37 | 1526.5 | 0.39 |

**Tables for Bravo (PSSERS 785/853 nm)**

| **Organism**  **(major carotenoid)** | **Raman bands positions from this study (cm^-1^)** | **Raman bands positions from literature (cm^-1^)** | **Reference** |
| --- | --- | --- | --- |
| ***Halobacterium* *salinarum* (bacterioruberin)** | 1505, 1150, 999 | 1505, 1150, 1000 | Jehlička et al., 2013a |
| ***Haloarcula marismortui***  **(bacterioruberin)** | 1506, 1150, 999 | 1509, 1155, 1002 | Oren et al., 2018 |
| ***Halorubrum sodomense***  **(bacterioruberin)** | 1506, 1150, 1000 | 1506, 1152, 1001 | Jehlička et al., 2013a |
| ***Salinibacter ruber***  **(salinixanthin)** | 1511, 1154, 1001 | 1512, 1155, 1003 | Jehlička et al., 2013a |
| ***Ectothiorhodosphira marismortui****  **(spirilloxanthin)** | 1509, 1150, 1001 | 1510, 1151, 1004 | Jehlička and Oren, 2013 |
| ***Dunaliella parva***  **(β–carotene)** | 1522, 1155, 1004 | 1525, 1157, 1005 | Jehlička et al., 2014a |
| ***Micrococcus luteus***  **(sarcinaxanthin)** | 1527, 1156, 1005 | 1529, 1156, 1005 | Jehlička et al., 2014a |
| ***Corynebacterium glutamicum***  **(decaprenoxanthin)** | 1523, 1156, 1006 | 1522, 1157, 1005 | Jehlička (unpublished materials) |
| ***** values taken from the wet pellets sample type | | |  |

**Comparison of Raman band positions for major carotenoids detected in this study (instrument RaPort 532 nm, sample type lyophilized cultures) and references in literature**
